# Non‐linear effect of sea ice: Spectacled Eider survival declines at both extremes of the ice spectrum

**DOI:** 10.1002/ece3.4637

**Published:** 2018-11-20

**Authors:** Katherine S. Christie, Tuula E. Hollmen, Paul Flint, David Douglas

**Affiliations:** ^1^ The Alaska SeaLife Center Seward Alaska; ^2^ The College of Fisheries and Ocean Sciences University of Alaska Fairbanks Fairbanks Alaska; ^3^ U.S. Geological Survey Alaska Science Center Anchorage Alaska; ^4^ U.S. Geological Survey Alaska Science Center Juneau Alaska

**Keywords:** Bering Sea, mark‐recapture, sea ice, *Somateria fischeri*, Spectacled Eider, survival

## Abstract

Understanding the relationship between environmental factors and vital rates is an important step in predicting a species’ response to environmental change. Species associated with sea ice are of particular concern because sea ice is projected to decrease rapidly in polar environments with continued levels of greenhouse gas emissions. The relationship between sea ice and the vital rates of the Spectacled Eider, a threatened species that breeds in Alaska and Russia and winters in the Bering Sea, appears to be complex. While severe ice can impede foraging for benthic prey, ice also suppresses wave action and provides a platform on which eiders roost, thereby reducing thermoregulation costs. We analyzed a 23‐year mark‐recapture dataset for Spectacled Eiders nesting on Kigigak Island in western Alaska, and tested survival models containing different ice and weather‐related covariates. We found that much of the variation in eider survival could be explained by the number of days per year with >95% sea ice concentration at the Bering Sea core wintering area. Furthermore, the data supported a quadratic relationship with sea ice rather than a linear one, indicating that intermediate sea ice concentrations were optimal for survival. We then used matrix population models to project population trajectories using General Circulation Model (GCM) outputs of daily sea ice cover. GCMs projected reduced sea ice at the wintering area by year 2100 under a moderated emissions scenario (RCP 4.5) and nearly ice‐free conditions under an unabated emissions scenario (RCP 8.5). Under RCP 4.5, stochastic models projected an increase in population size until 2069 coincident with moderate ice conditions, followed by a decline in population size as ice conditions shifted from intermediate to mostly ice‐free. Under RCP 8.5, eider abundance increased until 2040 and then decreased to near extirpation toward the end of the century as the Bering Sea became ice‐free. Considerable uncertainty around parameter estimates for survival in years with minimal sea ice contributed to variation in stochastic projections of future population size, and this uncertainty could be reduced with additional survival data from low‐ice winters.

## 1 INTRODUCTION

Climate change‐induced distributional shifts and extirpations have been documented for many animal taxonomic groups (Beever, Brussard, & Berger, 2003; Parmesan & Yohe, 2003; Pounds et al., 2006; Thomas & Lennon, 1999). The mechanisms causing these responses involve altered survival and reproduction as a result of changing environmental conditions (Croxall, Trathan, & Murphy, 2002; Post & Forchhammer, 2008; Saether et al., 2000). Predicting the response of populations to climate change is difficult, not only because it is challenging to predict the actual degree of environmental change, but also because the relationships between vital rates and environmental factors are often not known. To complicate matters, different vital rates such as reproduction and survival can respond in opposite directions to climate change (van de Pol et al., 2010), and species can have non‐linear responses to changing conditions (Ballerini, Tavecchia, Olmastroni, Pezzo, & Focardi, 2009; Stenseth & Mysterud, 2002).

For ice‐associated species occupying polar environments, changing conditions can impact vital rates (Barbraud, Weimerskirch, Barbraud, & Weimerskirch, 2000; Regehr, Hunter, Caswell, Amstrup, & Stirling, 2009). This is of particular concern because temperatures are warming at twice the global rate in the Arctic (IPCC, 2014), where rapid sea ice loss has recently occurred (Stroeve, Holland, Meier, Scambos, & Serreze, 2007). For many sea ice‐associated species, intermediate ice concentrations are ideal for population growth because open leads facilitate access to marine foods while the ice provides a platform for resting, denning, travel, and reproduction (Jenouvrier, Holland, & Stroeve, 2012; Kovacs, Lydersen, Overland, & Moore, 2011). The presence of sea ice also influences food availability in the marine ecosystem. Sea ice is associated with high productivity of phytoplankton, which in turn stimulates the production of krill and other zooplankton beneath the ice layer (Eiken, 2014). Sea ice also benefits the benthic invertebrate community by stimulating spring algal blooms that result in the deposition of organic material on the ocean floor (Eiken, 2014; Grebmeier et al., 2015). In light of these interdependencies of marine biota on sea ice, there is considerable concern about how sharp declines in sea ice will impact marine ecosystems (Ardyna et al., 2014; Meredith & King, 2005; Post et al., 2013; Serreze, Holland, & Stroeve, 2007; Stroeve et al.,^2007^).

**Figure 1:**
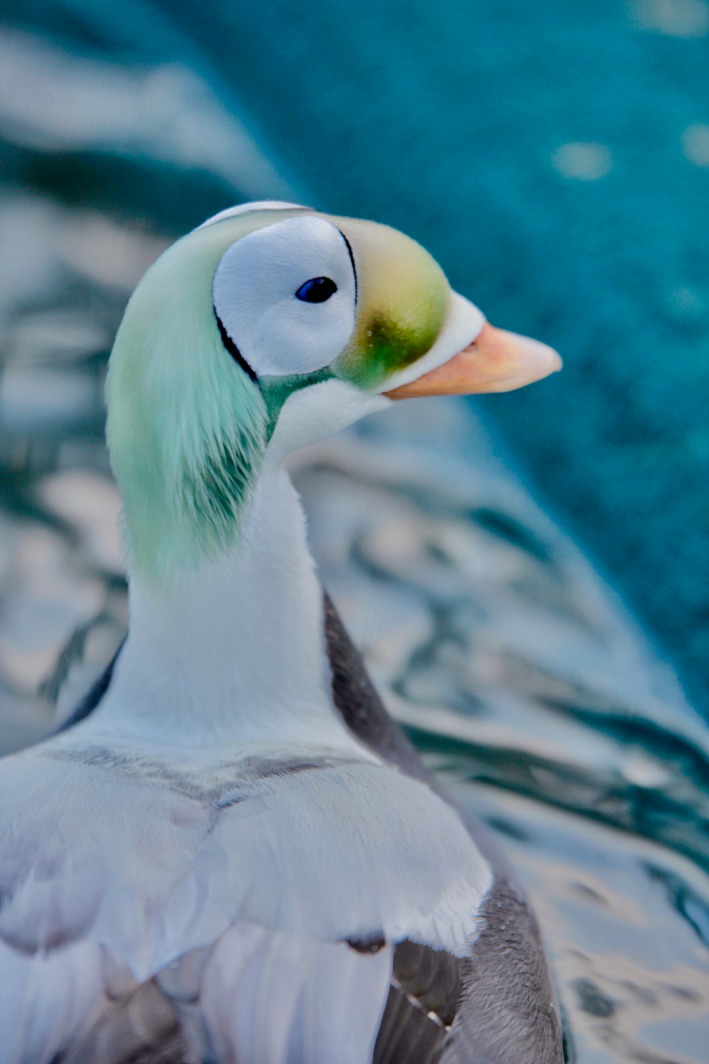
Spectacled Eider. Photo credit: The Alaska SeaLife Center

**Figure 2:**
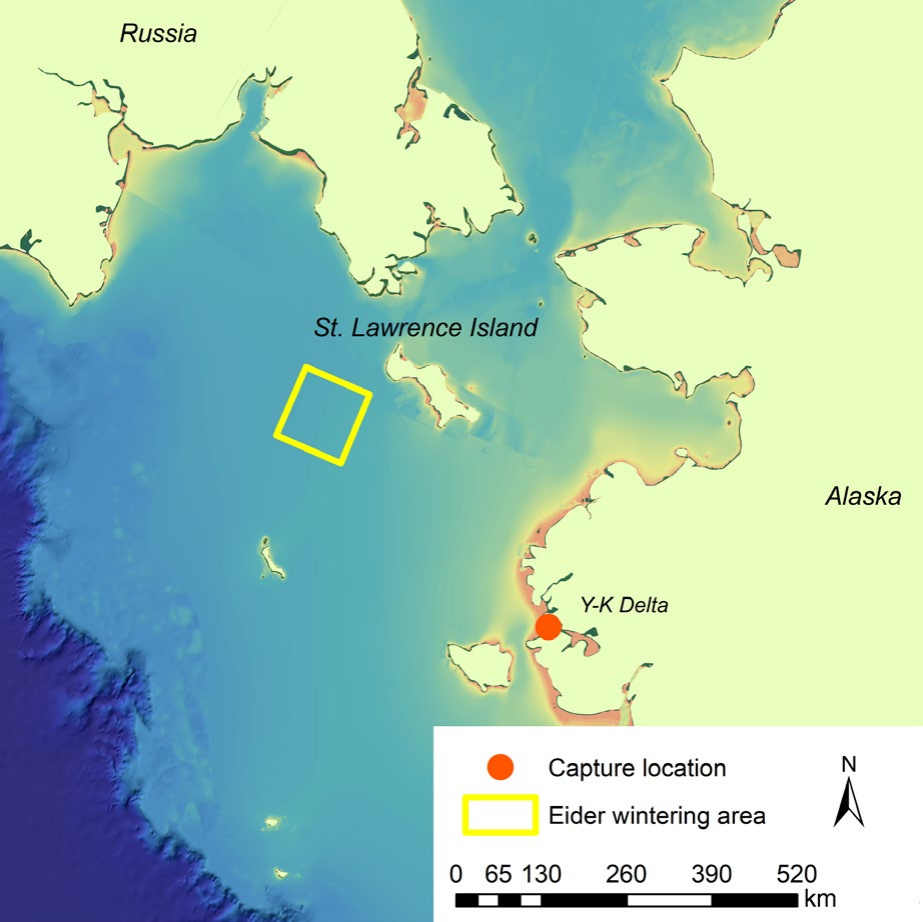
Map showing the Spectacled Eider wintering area in the Bering Sea and location of captures on Kigigak Island, Yukon‐Kuskokwim Delta

The Spectacled Eider (*Somateria fischeri*) is a diving sea duck that forages on bivalve mollusks in the benthic zone in Arctic waters (Petersen, Piatt, & Trust, 1998; Figure 1). Geographically distinct breeding populations occur in eastern Siberia, northern Alaska, and western Alaska; however, all three populations converge in a small wintering area south of St. Lawrence Island in the Bering Sea (Petersen, Larned, & Douglas, 1999; Figure 2). The area southwest of St. Lawrence Island is known to be a benthic hotspot with high microfaunal benthic biomass, the most abundant taxa being bivalves and polychaetes (Grebmeier et al., 2015). The bivalve species sampled in this region are thought to be the preferred size for Spectacled Eiders (12–30 mm), causing the St. Lawrence Island Polynya to stand out among other benthic hotspots in the Arctic in providing optimal foraging conditions for Spectacled Eiders (Grebmeier et al., 2015).

Spectacled Eiders experienced sharp population declines in western Alaska from the 1950s to 1990s, prompting their listing as “threatened” under the Endangered Species Act in 1993 (USFWS, 1993, 2010). Suspected causes for declines include exposure to lead shot on the breeding grounds, high predation rates, hunting pressure, and inhospitable sea ice conditions in the wintering area (Flint, Grand, & Petersen, 2016; Flint, Grand, Morse, & Fondell, 2000; Grand, Flint, Petersen, & Moran, 1998). Spectacled Eiders are closely associated with sea ice during winter (Petersen et al., 1999), which provides a platform for them to rest on between foraging bouts and dampens wave action, reducing the costs of thermoregulation (Lovvorn, Grebmeier, Cooper, Bump, & Richman, 2009). Extremely high sea ice concentrations, however, restrict access to preferred benthic foraging grounds (Lovvorn et al., 2014) and years with long periods of extensive sea ice have been associated with low survival (Flint et al., 2016). During periods of nearly continuous sea ice, eiders do not appear to disperse to other areas, and have been observed in very dense flocks (up to 10,000 birds) concentrated in small patches of open water (Petersen et al., 1999).

In this study, we analyzed 23 years of mark‐recapture data from Spectacled Eiders banded on their breeding grounds in Western Alaska, and assessed whether variation in survival could be explained by sea ice and weather conditions in the Bering Sea wintering area. A negative relationship between Spectacled Eider survival and years with high sea ice concentrations was documented by Flint et al., 2016 for the period 1992–2004. We were interested in repeating this analysis using a longer time series, and also testing for non‐linear relationships with sea ice, temperature, wind, and global atmospheric patterns. Subsequent to the survival analysis, we used an ensemble of Global Circulation Model (GCM) projections to estimate future sea ice conditions in the wintering area, and used those projections to model future Spectacled Eider population size under two different greenhouse gas emissions scenarios.

## 2 MATERIALS AND METHODS

### 2.1 Study site

The mark‐recapture study was conducted on Kigigak Island (32.5 km^2^, 60°50*'*N, 165°50*'*W) on the coast of the Yukon‐Kuskokwim Delta (hereafter “Y‐K Delta”) of western Alaska (Figure 2). Kigigak Island supports a multitude of productive ponds, lakes, and tidally influenced sloughs, and hosts large concentrations of breeding waterfowl including geese, ducks, and swans. Vegetation consists of tidally influenced sedge meadows and upland tundra vegetation including lichens, mosses, forbs, grasses, and dwarf shrubs. The climate is sub‐Arctic, with mean temperatures ranging from a high of 17.5°C in July to an average low of −17.3°C in January (U.S. Climate Data Center 2017). The Spectacled Eider population on Kigigak Island and nearby sites on the mainland have been studied in detail for over two decades (Ely, Dau, & Babcock, 1994; Flint et al., 2016; Flint, Morse, Grand, & Moran, 2006; Grand & Flint, 1997; Grand et al., 1998).

### 2.2 Field methods

For this study, we focused on female Spectacled Eiders, which show strong site fidelity to breeding areas. On Kigigak Island, distances between old and new nests were within 2.2 km (Moran, 2000). Therefore, we made the assumption that there was no permanent emigration by females from the study area. The core nesting area for Spectacled Eiders on the Y‐K Delta was defined based on medium to high densities of nesting birds detected during aerial surveys from 1988 to 1994. Kigigak Island falls within this core nesting area, and approximately 70% of the island was searched for Spectacled Eider nests each year. Nesting habitat consisted primarily of high graminoid and intermediate sedge meadow (Grand et al., 1998). Incubating female Spectacled Eiders were located by searching variable numbers of 412 × 412 m plots each May and June from 1992 to 2015. A sample of adult females were captured using manually triggered bow‐nets or mist‐nets. All capture activities were conducted 1–4 days prior to expected hatch to minimize the chance of nest abandonment. Expected hatch date was examined using float angles of three eggs per nest (Westerkov, 1950). Upon capture, adult Spectacled Eider females were marked with U.S. Geological Survey metal tarsal bands and numbered yellow plastic tarsal bands or nasal discs (Lokemon & Sharp, 1985). Nasal discs were introduced to facilitate resighting of marked birds, therefore, nasal‐tagged birds may have had higher resighting probabilities than those without. However, concerted efforts were made to view the legs of all birds encountered to maximize the probability of tag identification. Previously, banded females were identified visually using scopes and binoculars and were only recaptured if marks were unreadable. Field crews searched for broods over a 5‐day period in mid‐July. Ducklings were captured and banded with both metal and plastic bands at approximately 25–35 days of age.

### 2.3 Survival analysis

A maximum likelihood approach using Cormack‐Jolly‐Seber models was used to analyze the mark‐recapture data and estimate apparent survival (*φ*) and resighting probability (*p*; Lebreton, Burnham, Clobert, & Anderson, 1992) using Program MARK and a logit‐link function (White & Burnham, 1999). We used an information theoretic approach to assess the relative explanatory ability of different models of *p* and *φ*, and used an AIC corrected for small sample size and overdispersion (QAIC_c_). AIC scores were adjusted for overdispersion using an overdispersion parameter (ĉ), taken as the ratio of deviance between real and simulated data. We estimated ĉ using the median ĉ method in Program MARK (White & Burnham, 1999), based on the model *p*(year + age)*φ*(year + age). We modeled *p* as a function of year, age, nest success, year + age, year*age, age + nest success, and as a constant (See Supporting Information Table S1 for full list of models). We considered three age classes: ducklings (age 30 days to 1 year), second‐year birds (1–2 years), and adults. Birds did not return to the breeding grounds until they were 2 years of age, and we assumed survival from 1 to 2 years of age was equal to adult survival. We were able to estimate *p* for second‐year birds and adults, and held *p* constant at 0 for first‐year birds. Mean annual nest success at Kigigak Island was included as a covariate because in years of low nest success, failed nesting females left the study area mid‐breeding season and hence were difficult to resight. We did not model *p* as a function of tag type (tarsal vs. nasal) because this was confounded with age and year, and individual birds often had both types of tags within their lifetime.

For survival, we tested for differences among years and age classes; however, we were most interested in the potential relationships between survival and annually varying ice, temperature, and wind conditions in the Bering Sea, and global atmospheric patterns such as the Arctic Oscillation (AO) and Pacific Decadal Oscillation (PDO). Annual indices of AO, PDO, mean winter sea surface temperature, and a measure of winter North‐South wind anomalies in the Bering Sea were downloaded from the National Oceanic and Atmospheric Administration's Bering Climate data portal (https://www.beringclimate.noaa.gov/data/index.php). The four 25 × 25 km cells defining the core wintering area were originally identified by (Petersen & Douglas, 2004) based on utilization distributions of 13 satellite‐tagged individuals from 1993 to 1997, confirmed by aerial surveys of overwintering Spectacled Eiders. Observed sea ice concentrations were extracted for the core wintering area from daily gridded (25 km resolution) estimates that were derived from passive microwave satellite imagery using the Bootstrap Algorithm and disseminated by the National Snow and Ice Data Center, Boulder, CO (Comiso, 2017). The core wintering area spanned four grid cells, and the ice concentration for a given day was defined as the minimum among the four cells. We calculated number of days from November through April with >95% ice cover (hereafter, “extreme ice days”), and an index of sea ice severity based on the number of consecutive days with heavy sea ice (Flint et al., 2016). For this index, we counted the number of consecutive days with >95% sea ice cover, allowing for 1‐day drops below 95%. Isolated one‐day events of high sea ice cover were not counted. The ice index accounted for both the length of high‐ice periods and the frequency of these periods for each year. We used the following equation to calculate the index (*I*; Flint et al., 2016):

$$I=\sum_{i=I}^{B}D\times \text{ln}\left(D\right)$$

where *B* is the total number of periods within a year and *D* is the number of days within each period. According to this equation, years with lengthy contiguous periods of high sea ice cover will have a higher ice index. For all climate covariates except for wind anomaly, quadratic terms were considered to test for non‐linear effects. Models with all combinations of climate and sea ice covariates were tested (see Supporting Information Table S2 for full list of models). Because models that replace time‐varying survival with a covariate derived estimate tend to have large differences in numbers of parameters, we conducted an Analysis of Deviance (ANODEV), which provides a means of assessing whether a covariate explains a significant amount of deviance (Skalski, Hoffman, & Smith, 1993).

### 2.4 Climate model data

We obtained projections of daily sea ice concentration from archives of CMIP5 GCM outputs (see Supporting Information Table S3 for a list of models). For each model, we extracted data from 2 to 6 grid cells (depending on the spatial resolution of the model output) that fell within the core wintering area, and took the average sea ice cover from all cells. Because our data requirements were specific (daily sea ice concentrations from 1950 to 2100), our ensemble was limited by data availability to eight models. Historical data were necessary to compare modeled simulations of sea ice concentrations to observed conditions dating back to 1950. For projected sea ice concentrations, we used model outputs based on two emissions scenarios: Representative Concentration Pathway (RCP) 4.5 and RCP 8.5, in which radiative forcing above preindustrial levels stabilizes near 4.5 W/m^2^ by 2100 under RCP 4.5, and reaches 8.5 W/m^2^ on an increasing trajectory by 2100 under RCP 8.5. From the GCM daily sea ice concentration outputs, we tallied the number of extreme ice days (days with >95% cover) and number of minimal sea ice days (days with <15% ice cover) at the Spectacled Eider wintering area from November through April each year. We chose the 95% cutoff to represent days with contiguous ice cover, which limits access by eiders to benthic foraging grounds (Lovvorn et al., 2014). We chose the 15% cutoff to represent the opposite scenario, where sea ice is largely absent (Wang & Overland, 2015). A weighted mean across models was calculated based on comparisons to observations using methods developed by Knutti et al. (2017). Briefly, this weighting scheme is based on model performance and interdependence, and is calculated as follows:

$$w_{i}=\frac{e^{-\frac{D_{i}^{2}}{\sigma_{D}^{2}}}}{\left(1+\sum_{j\neq i}^{M}e^{-S_{\mathrm{ij}}^{2}}/\sigma_{s}^{2}\right)}$$

where *D_i_* is the root‐mean‐squared distance between observations and projections, and *S_ij_* is the root‐mean‐squared distance between model *i* and model *j*. *σ_D_* and *σ_s_* are constants that determine how strongly models are weighted by performance or interdependence, respectively. As *σ_D_* increases, only models with projections that are extremely similar to observed values receive any weight. As *σ_s_* increases, increasingly distant models are considered to be similar, and as *σ_s_*→∞, the weighting scheme begins to have no effect. To balance over‐ and under‐weighting models, we calculated *σ_D_* as the mean root‐square distance between models and observed data (passive microwave satellite imagery for the core wintering area), and *σ_s_* as the mean root‐square distance between all model pairs (Sanderson, Knutti, & Caldwell, 2015).

### 2.5 Ice projections and stochastic population modeling

Given the non‐linear effect of sea ice on survival, the issue becomes how to predict survival in both high and low sea ice years such that the estimated effect in a minimal ice year is not an artifact of extrapolation of an extreme ice effect. There is a clear negative correlation in the number of extreme and minimal ice days when viewed across years. To calculate survival of Spectacled Eiders based on projected future ice conditions, we first estimated the effects of observed extreme and minimal sea ice conditions on eider survival separately. We estimated the relationship between eider survival and the number of model simulated days per year with ice concentrations >95% that had an effect on eider survival. To do this, we used QAICc to select among models with different thresholds of sea ice days from 20 to 100 extreme ice days per year (representing observed conditions). Years that met this threshold had a covariate value for number of extreme ice days, and years that did not meet this threshold had the covariate value set at 0. Similarly, for minimal sea ice conditions, we used QAICc to select the model with the appropriate threshold of minimal sea ice days from 40 to 110 days per year with sea ice concentrations <15%. Years that met this threshold had a covariate value for number of days with low ice concentrations, and years that did not meet this threshold had the covariate value set at 0. Thus, extreme and minimal ice days became pseudo‐dummy variables in that no year would have a positive covariate value for both. Using this method, we were able to create a single model that estimated the relationship between survival and each end of the spectrum of ice concentrations. We then used parameter estimates from this model to estimate survival under projected future sea ice conditions.

We projected future population size of female Spectacled Eiders on Kigigak Island using stochastic matrix population models (Caswell, 2001; Morris & Doak, 2002). We used a pre‐breeding census that included three age classes: juveniles, second‐year birds, and after second‐year birds. Breeding propensity, mean nest success, fecundity, and chick survival at Kigigak Island were obtained from Fischer, Williams, & Stehn, 2017 and Flint et al., 2016, and fertilities were calculated as the product of these vital rates and juvenile survival. Survival probabilities were estimated for each year between 2016 and 2100 using the ice threshold model described above and GCM sea ice projections. For each of 1,000 iterations, we drew a random number from a beta distribution of survival probabilities using the survival estimate and associated variance at each projected time step. Fertilities were drawn from a stretched beta distribution using the mean and variance based on historical observations at Kigigak Island. We used a starting population size of 127 female Spectacled Eiders (Flint et al., 2016).

## 3 RESULTS

From 1992 to 2015, we obtained encounter histories for 1,328 birds, of which 649 were initially captured as ducklings. We observed slight overdispersion in the data, and ĉ was estimated at 1.326. For detection probability, the model that received the most support allowed detection to vary by year and age, and the relative likelihood of this model compared to other models was 1.0 (Table 1). This parameterization of *p* was used in subsequent models of *φ* (survival)

**Figure 3:**
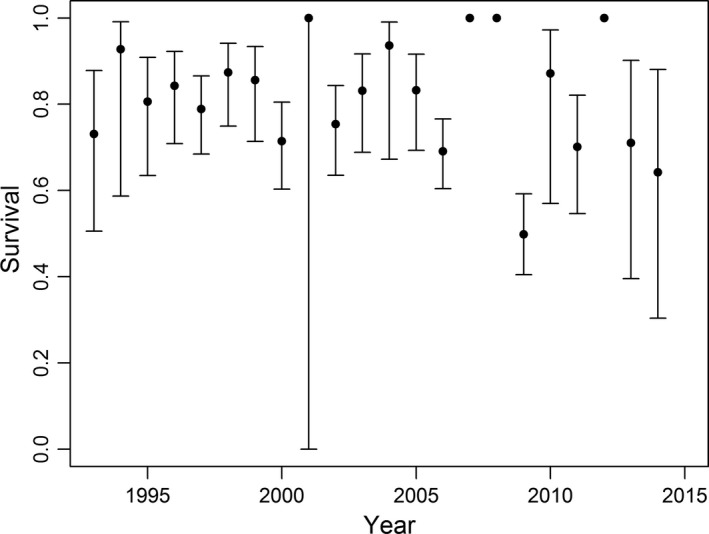
Annual survival of adult female Spectacled Eiders nesting at Kigigak Island, Alaska

. For *φ* (survival), the best approximating model allowed survival to vary annually for adults but not first‐year birds, for which survival was modeled as a constant (QAIC_c_ *ω* = 0.84; Table 1). Annual survival of adult females ranged from 0.50 ± 0.05 to 1.00 ± 0.00 (Figure 3), and mean first‐year survival was 0.22 ± 0.02. The second best‐approximating model included an age*year interaction term and had a substantially lower model likelihood (QAIC_c_ *ω* = 0.14). The third best‐supported model indicated a quadratic relationship between survival and number of extreme ice days per year (days with >95% sea ice cover).

**Table 1:** Models of detection probability and survival for Spectacled Eiders captured on Kigigak Island

Model	ΔQAIC_c_	QAIC_c_ Weight	Num. Par	QDeviance	Deviance explained (%)
Detection					
*p*(year + age) *φ*(.)	0.00	1.00	25	4,521.70	—
*p*(year*age) *φ*(.)	29.43	0.00	47	4,505.92	—
*p*(nest success + age) *φ*(.)	31.82	0.00	4	4,595.99	67.28
*p*(year) *φ*(.)	114.88	0.00	24	4,638.61	—
*p*(nest success) *φ*(.)	157.77	0.00	3	4,723.94	43.38
Survival					
*p*(year + age) *φ*(year, adults only)	0.00	0.84	47	4,274.36	—
*p*(year + age) *φ*(year*age)	3.56	0.14	70	4,229.84	—
*p*(year + age) *φ*(ice days^2^ + age)	9.22	0.01	28	4,322.68	44.25
*p*(year + age) *φ*(year + age)	9.57	0.01	46	4,286.00	—
*p*(year + age) *φ*(SST^2^*age)	11.78	0.00	30	4,321.14	30.00

Note

Total between‐year variation explained by covariates was calculated with the Analysis of Deviance Test (ANODEV). See Supporting Information Tables S1 and S2 for full suite of models.

**Figure 4:**
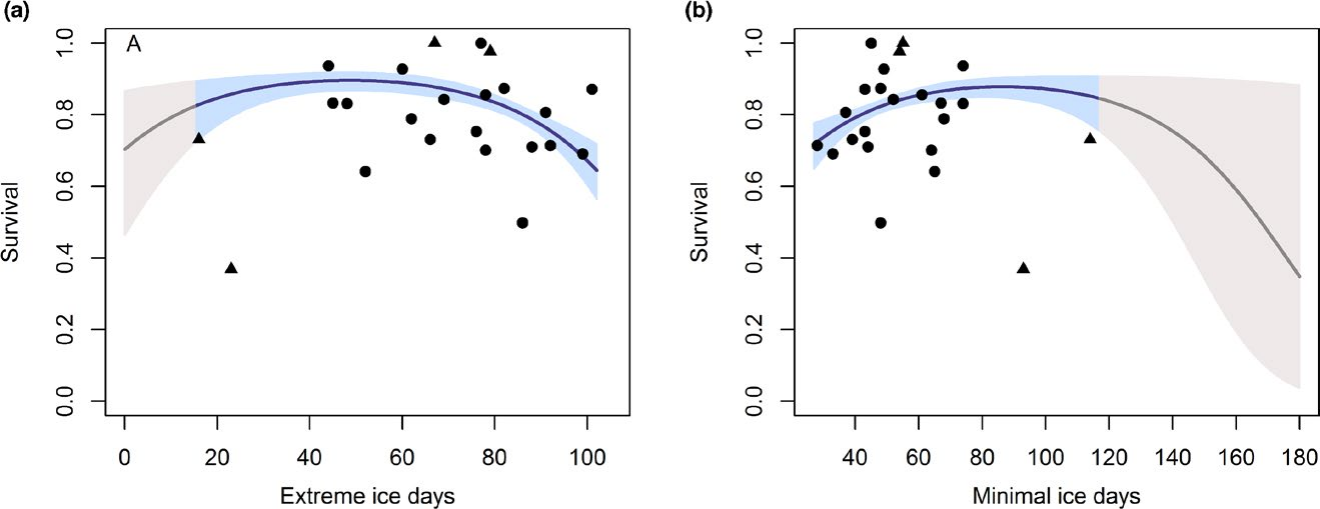
Survival and 95% confidence intervals of adult female spectacled eiders as a function of (a) extreme sea ice conditions at the wintering area (number days per year with sea ice concentrations >95%) and (b) minimal ice conditions (number of days per year with sea ice concentrations <15%). The gray line and shading represents predicted survival under sea ice conditions lower than observed during our study period. Black circles indicate survival estimates from the model *p*(year + age)*φ*(year, adults only) and black triangles denote survival estimates from the simpler model *p*(age)*φ*(year, adults only). Both models were used because survival for certain years was not estimated in the fully time‐parameterized model

To test whether the ice covariates explained a significant amount of variation in survival, we conducted an Analysis of Deviance (ANODEV). The quadratic term for extreme ice days explained 44% of total deviance based on ANODEV test results (*F* = 7.14, *p* = 0.005, Table 1). In comparison, the linear relationship with ice explained only 24% of total deviance (*F* = 1.20, *p* = 0.47). Parameter estimates from our model indicated that survival increased with number of extreme ice days until approximately 52 days, beyond which survival flattened out and then began to decrease at approximately 80 days (Figure 4a). For comparison, we modeled survival as a function of minimal ice days (days with sea ice concentrations <15%). Survival increased with minimal ice days until approximately 90 days, and then declined, although confidence intervals were wide, indicating considerable uncertainty (Figure 4b).

**Figure 5:**
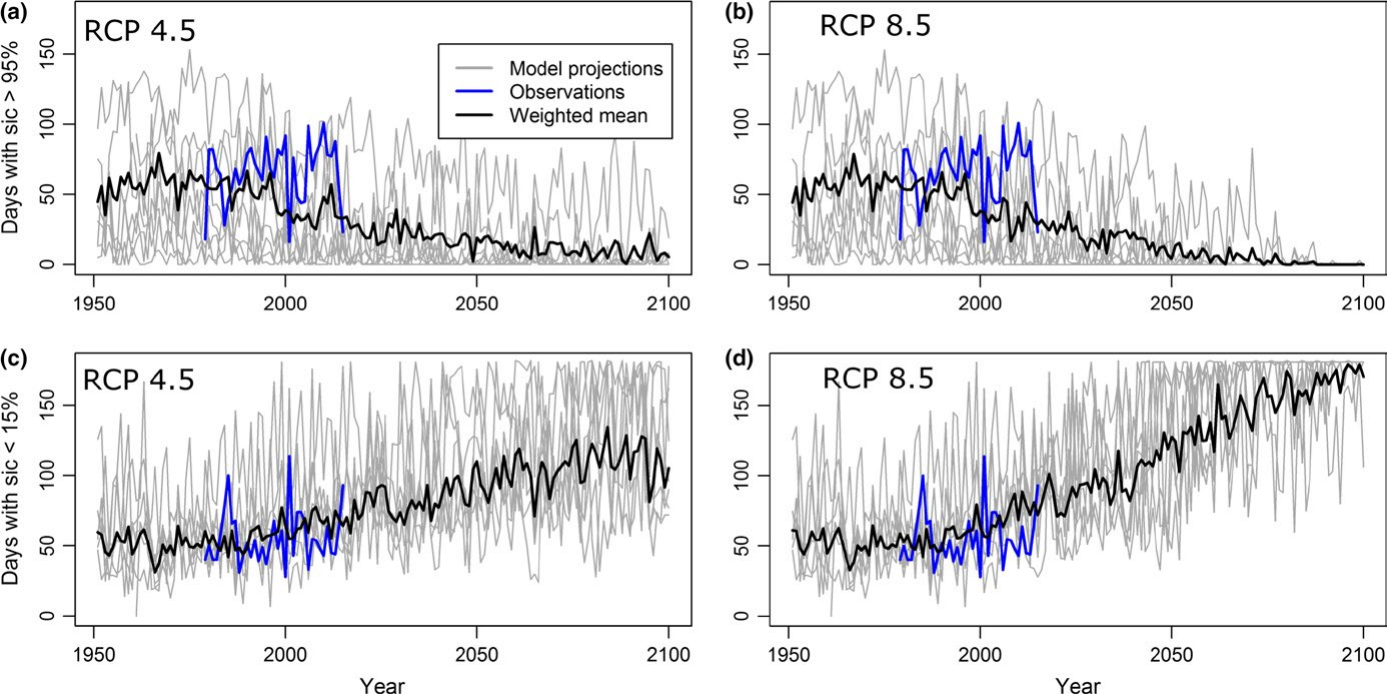
Projected number of days during November‐April (181 days total) with sea ice cover >95% (a and b) and days with <15% sea ice cover (c and d) based on eight CMIP5 General Circulation Models (gray), their weighted mean (black), and observations (blue)

All eight GCMs projected steadily increasing numbers of ice‐free days toward the end of the 21st century, increasing more quickly for RCP 8.5 than RCP 4.5 (Figure 5). For RCP 8.5, the Spectacled Eider wintering area was projected to have over 100 minimal ice days by 2040 and be virtually ice free (175 out of 181 winter days without significant ice) by 2100 according to weighted estimates (Figure 5). For RCP 4.5, the eider wintering area was projected to have at least 100 minimal ice days by the end of the century. This compares with a mean of 56 minimal ice days per year during our study period in the core wintering area.

The survival analysis in MARK indicated that survival declined when there were more than 80 extreme ice days per year or when there were more than 100 minimal ice days per year (Figure 4). The negative relationship was stronger for extreme ice days (*β*_extreme_ = −0.901, 95% CI = −1.177, −0.626) than for minimal ice days (*β*_minimal_ = −0.178, 95% CI = −0.364, 0.007). However, caution must be used in interpreting projected survival rates because 95% confidence intervals around the parameter estimate for minimal ice days slightly overlapped zero. Using this relationship to project survival rates, we found that survival under moderate sea ice conditions (when threshold for extreme ice and minimal ice days were not met) was relatively high at 0.88. These conditions were projected to occur early in the century, prior to 2049 for RCP 4.5, and prior to 2040 for RCP 8.5 (Figure 5). For the last half of the century, mean survival decreased to 0.84 for RCP 4.5 and 0.76 for RCP 8.5 in 2100.

**Figure 6:**
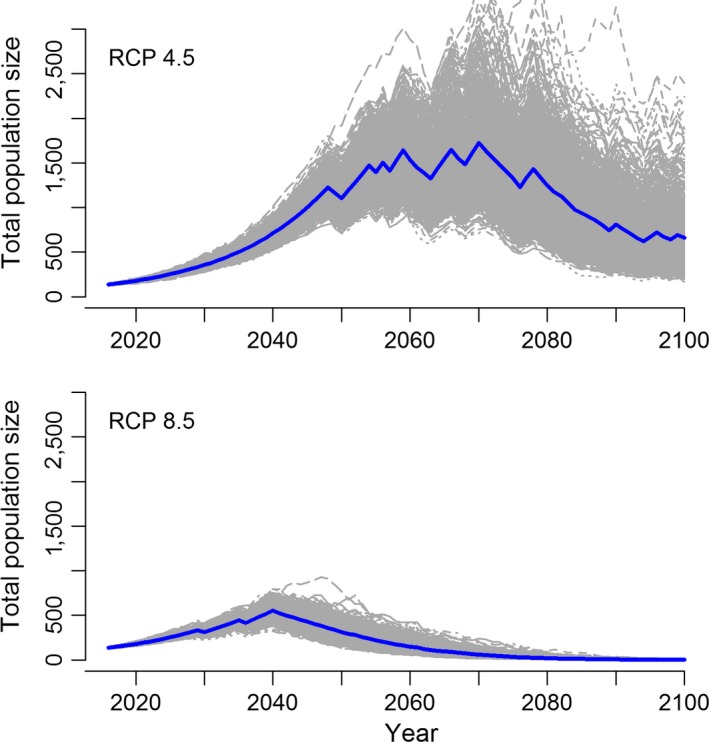
Stochastic projections of future population size of Spectacled Eiders on Kigigak Island under RCP 4.5 (top) and RCP 8.5 (bottom). Each gray line represents a single iteration of the matrix population model, and the blue line represents the mean of all 1,000 iterations

Stochastic population projections demonstrated that under RCP 4.5, population size increased to 1,728 ± 428 (*SD*) females in 2069 coincident with moderate ice conditions early and mid‐century (Figure 6). However, as conditions on the Bering Sea became increasingly ice‐free toward the end of the century, the Kigigak population began to decline. It should be noted that there was a large amount of variation in stochastic projections for RCP 4.5 due to the combination of rapid population growth mid‐century and high uncertainty around parameter estimates for survival under future sea ice conditions. For RCP 8.5 projections, eider population size increased to a mean of 553 ± 74 females in 2041 and decreased to a mean of 2 ± 1 females in 2100 (Figure 6).

## 4 DISCUSSION

This study showed that much of the variation observed in Spectacled Eider survival from 1993 to 2015 could be explained by sea ice conditions at the species’ wintering area in the Bering Sea. Survival was high in years with moderate sea ice, and decreased in winters with long periods of heavy sea ice and also in winters with very little sea ice. This tendency of vital rates to peak under moderate sea ice conditions has been observed in other sea ice‐associated birds such as Terre Adélie Emperor Penguins (*Aptenodytes forsteri*; Jenouvrier et al., 2012) and Antarctic Snow Petrels (*Pagodroma nivea*; Barbraud et al., 2011). However, this is the first time a non‐linear relationship with sea ice has been detected in Spectacled Eiders, for which only linear relationships have been tested previously (Flint et al., 2016; Petersen & Douglas, 2004).

The mechanisms for how eiders are affected by severe sea ice conditions are fairly well‐understood. Although Spectacled Eiders are adapted to harsh winter conditions, severe ice restricts access to benthic foraging grounds and can lead to high mortality rates and population declines (Flint et al., 2016; Petersen & Douglas, 2004). It is possible that eiders moved out of the core wintering area in years of dense sea ice. However, eiders are thought to cease foraging and subsist on reserves rather than move to more distant areas, similar to other duck species (Lovvorn, 1994; Suter & Van Eerden, 1992). This assumption is supported by observations of dense aggregations of Spectacled Eiders in the core wintering grounds in years when extremely high ice concentrations prevented them from accessing preferred foraging areas (Lovvorn et al., 2014). These dense flocks that occur in open leads are unlikely to deplete food resources due to the constant movement of the ice, which provides access to new food resources over the course of the winter (McDonald, Feder, & Hoberg, 1981; Stoker, 1981). It is possible that eiders were able to forage in small leads in the ice during times when ice cover was greater than 95%. Unfortunately, our sea ice data were not high enough resolution to detect these fine‐scale openings and small leads in the ice. Despite this limitation, and the dynamic nature of sea ice, our models indicated a decline in survival in years with heavy sea ice, even when measured at the 25‐km scale.

We hypothesize that the relationship between Spectacled Eider survival and low‐ice conditions can be explained in large part by energetics. The energetic cost in aquatic birds of having full contact with water (swimming) is substantially greater than having no contact (roosting; De Vries & Van Eerden, 1995). For Spectacled Eiders, respiratory studies on captive birds indicate that the cost of thermoregulation is reduced when birds have access to a platform to rest on between foraging bouts (Lovvorn et al., 2009). Therefore, when ice is present, eiders can survive with less food because their energetic demands are lower. Ice cover also suppresses wave height, providing a favorable environment for feeding (Divoky, 1981), and can passively transport eiders to new foraging areas within the core wintering area (Petersen & Douglas, 2004). Lastly, the presence of sea ice in the Bering Sea is linked to spring algal blooms, which cause carbon to be deposited in the benthic zone, in turn enhancing biological productivity (Brown & Arrigo, 2013; Cooper et al., 2012). Therefore, moderate amounts of ice may be a key habitat feature that reduces energetic costs of Spectacled Eiders, dampens wave action, and increases the productivity of benthic feeding areas. Exactly how much ice is required to achieve these conditions is unknown. We defined “minimal ice cover” as concentrations below 15%, as defined by Wang and Overland (2015); however, it is possible that higher concentrations are needed to provide suitable overwintering conditions for eiders.

Our models showed a great deal of uncertainty associated with the relationship between survival and minimal sea ice conditions. Therefore, we recommend that additional years of survival data will be necessary to test our hypothesis that low sea ice conditions negatively impact survival. This uncertainty is in large part due to our time series of observations not containing many years of minimal sea ice; only one year fell above the 100‐day threshold for minimal ice conditions, whereas 7 years fell above the 80‐day threshold for extreme ice conditions. In recent history, Spectacled Eider populations were probably limited primarily by dense ice pack rather than low sea ice cover on the Bering Sea. Additional data on eider survival in low‐ice years will decrease uncertainty about this relationship. We also acknowledge the uncertainty associated with projecting survival in response to future sea ice conditions that are expected to be lower than previously observed. Projections of wildlife responses to future environmental conditions are challenging due to the complexity of natural systems and the omission of important drivers like biotic interactions (Allen, Stott, Mitchell, Schnur, & Delworth, 2000; Pearson & Dawson, 2003).

According to GCM projections, the Bering Sea core wintering area is likely to experience a decline in sea ice concentrations over the next several decades that may result in a shift in the factors limiting Spectacled Eider populations. We acknowledge that GCMs project sea ice at a fairly coarse resolution relative to the size of the Spectacled Eider wintering area. Our goal was to understand, broadly, how sea ice concentrations are expected to change in the region that is important to overwintering eiders. GCMs projected minimal ice days to increase from an average of 56 days during our study period (1992–2015) to approximately 100 at the end of the century under RCP 4.5 and 175 days under RCP 8.5. These results agree with a CMIP5 GCM study of future sea ice cover in the Alaskan Arctic (Wang & Overland, 2015) that reported projections of ice‐free waters in the Bering Sea by the end of the century under RCP 8.5. Contingent on projections of sea ice, estimated Spectacled Eider survival remained fairly high (0.88) until mid‐century and then declined to 0.82 (RCP 4.5) and 0.77 (RCP 8.5) in 2100. Stochastic population models indicated that these changes in survival have pronounced effects on eider population size. Projections differed markedly between the two climate change scenarios, with a net increase in population size in 2100 under RCP 4.5 due to moderate conditions favoring high survival until 2069 versus a decline to near extirpation by 2100 under RCP 8.5. These stochastic population models resulted in highly variable population estimates due to the incorporation of two major sources of uncertainty: (a) uncertainty associated with the relationship between eider survival and minimal ice days, and (b) uncertainty about future sea ice conditions, resulting in dramatically different outcomes for RCP 4.5 and 8.5. Additional survival data from years with minimal ice cover and further refinement of GCM models and will reduce this uncertainty.

Climate change has the potential to impact Spectacled Eider winter ecology through changes in both sea ice concentrations and prey species composition. This paper examines the former, but changing prey densities in the Bering Sea may be equally, if not more important. It has been hypothesized that the concentration of wintering Spectacled Eiders in the St. Lawrence Island polynya is a result of abundant prey taxa (bivalves) and prey size (12–30 mm) relative to other locations in the Arctic (Grebmeier et al., 2015). Grebmeier, 2012 found that interannual variability in sea ice in the Bering Sea is correlated with changes in benthic ecosystem structure and invertebrate species composition. Multiple consecutive years of low sea ice in the Bering Sea could conceivably lead to a major ecosystem shift from marine communities dominated by high densities of benthic macro‐invertebrates toward communities dominated by demersal fish and predatory invertebrates (Grebmeier et al., 2006). This in turn would be incompatible with the Spectacled Eider's requirement for high benthic invertebrate prey abundance.

Many species respond to climate change by shifting their ranges (Chen, Hill, Ohlemüller, Roy, & Thomas, 2011; Erb, Ray, & Guralnick, 2011; Hitch & Leberg, 2007; Parmesan & Yohe, 2003). If a species must respond to changing climatic conditions but is unable to shift its distribution due to barriers to migration, the inability to move, or the absence of alternative habitat, then populations are likely to go extinct (Beever et al., 2003; Durance & Ormerod, 2010; Sinervo et al., 2010). Spectacled Eiders shifted their core molting areas up to 90 km over two decades (Sexson et al., 2016), and could conceivably shift their wintering area in a similar manner. The establishment of a new wintering area further north would require (a) the behavioral plasticity to make such a shift, and (b) suitable prey densities in a location with optimal ice conditions to support the population.

## CONFLICT OF INTEREST

None declared.

## AUTHOR CONTRIBUTIONS

KC, PF, and TH conceived of the study. KC analyzed the data and wrote the manuscript. PF helped analyze and interpret results of the survival analysis. DD provided historical sea ice data and helped with the sea ice analysis and interpretation of results. All authors edited drafts of the manuscript and gave final approval for publication.

## DATA ACCESSIBILITY

All survival and ice data used for this publication is publicly available in Dryad Digital Repository (https://datadryad.org/), https://doi.org/10.5061/dryad.s1c5m5k.
