# Supplementary material for: Non‐linear effect of sea ice: Spectacled Eider survival declines at both extremes of the ice spectrum
Source: Ecol Evol. 2018 Nov 20;8(23):11808–18. doi: 10.1002/ece3.4637 (PMC6303746; doi:10.1002/ece3.4637)
Supplement: Supplementary file 1 [file ECE3-8-11808-s001.docx]

**Supporting Information**. Model selection results for detection probability and survival of Spectacled Eiders and institute list for CMIP5 General Circulation Models.

Table S1. Model selection results for detection probability of Spectacled Eiders

| Model | QAICc | ΔQAIC_c_ |  | QAIC_c_ Weight | Num. Par | QDeviance |
| --- | --- | --- | --- | --- | --- | --- |
| p(year+age) Ψ(.) | 4572.18 | 0.00 | 1.00 | 1.00 | 25 | 4521.70 |
| p(year*age) Ψ(.) | 4601.62 | 29.43 | 0.00 | 0.00 | 47 | 4505.92 |
| p(nest success+age) Ψ(.) | 4604.00 | 31.82 | 0.00 | 0.00 | 4 | 4595.99 |
| p(year) Ψ(.) | 4687.06 | 114.88 | 0.00 | 0.00 | 24 | 4638.61 |
| p(nest success) Ψ(.) | 4729.95 | 157.77 | 0.00 | 0.00 | 3 | 4723.94 |
| p(age) Ψ(.) | 4754.82 | 182.63 | 0.00 | 0.00 | 3 | 4748.81 |
| p(.) Ψ(.) | 4882.90 | 310.71 | 0.00 | 0.00 | 2 | 4878.89 |

Table S2. Model selection results for survival of Spectacled Eiders.

| Model | QAICc | ΔQAIC_c_ | QAIC_c_ Weight | Num. Par | QDeviance |
| --- | --- | --- | --- | --- | --- |
| p(year+age) Ψ(year adults) | 4370.06 | 0.00 | 0.84 | 47 | 4274.36 |
| p(year+age)Ψ(year*age) | 4373.62 | 3.56 | 0.14 | 70 | 4229.84 |
| p(year+age)Ψ(ice days^2^+age) | 4379.29 | 9.22 | 0.01 | 28 | 4322.68 |
| p(year+age)Ψ(year+age) | 4379.63 | 9.57 | 0.01 | 46 | 4286.00 |
| p(year+age)Ψ(SST^2^*age) | 4381.84 | 11.78 | 0.00 | 30 | 4321.14 |
| p(year+age)Ψ(ice days^2^*age) | 4382.39 | 12.32 | 0.00 | 30 | 4321.69 |
| p(year+age)Ψ(ice index^2^+age) | 4387.55 | 17.48 | 0.00 | 28 | 4330.94 |
| p(year+age)Ψ(SST^2^+age) | 4388.73 | 18.66 | 0.00 | 28 | 4332.12 |
| p(year+age)Ψ(ice days*age) | 4389.84 | 19.78 | 0.00 | 28 | 4333.23 |
| p(year+age)Ψ(ice days+age) | 4390.50 | 20.43 | 0.00 | 27 | 4335.93 |
| p(year+age)Ψ(ice index^2^*age) | 4390.74 | 20.68 | 0.00 | 30 | 4330.05 |
| p(year+age)Ψ(ice index+age) | 4391.85 | 21.79 | 0.00 | 27 | 4337.29 |
| p(year+age)Ψ(ice index*age) | 4393.89 | 23.83 | 0.00 | 28 | 4337.28 |
| p(year+age)Ψ(mean ice^2^+age) | 4394.37 | 24.31 | 0.00 | 28 | 4337.76 |
| p(year+age)Ψ(AO^2^*age) | 4394.54 | 24.47 | 0.00 | 30 | 4333.84 |
| p(year+age)Ψ(mean ice^2^*age) | 4394.54 | 24.48 | 0.00 | 30 | 4333.85 |
| p(year+age)Ψ(SST*age) | 4395.53 | 25.47 | 0.00 | 28 | 4338.92 |
| p(year+age)Ψ(AO^2^+age) | 4396.19 | 26.13 | 0.00 | 28 | 4339.59 |
| p(year+age)Ψ(mean ice*age) | 4398.08 | 28.02 | 0.00 | 28 | 4341.48 |
| p(year+age)Ψ(AO*age) | 4398.23 | 28.17 | 0.00 | 28 | 4341.62 |
| p(year+age)Ψ(SST+age) | 4398.76 | 28.70 | 0.00 | 27 | 4344.20 |
| p(year+age)Ψ(mean ice+age) | 4398.92 | 28.85 | 0.00 | 27 | 4344.35 |
| p(year+age)Ψ(PDO+age) | 4399.10 | 29.03 | 0.00 | 27 | 4344.53 |
| p(year+age)Ψ(PDO*age) | 4400.64 | 30.58 | 0.00 | 28 | 4344.03 |
| p(year+age)Ψ(PDO^2^+age) | 4401.14 | 31.07 | 0.00 | 28 | 4344.53 |
| p(year+age)Ψ(PDO^2^*age) | 4401.74 | 31.68 | 0.00 | 30 | 4341.05 |
| p(year+age)Ψ(AO+age) | 4402.26 | 32.19 | 0.00 | 27 | 4347.69 |
| p(year+age) Ψ(age)) | 4404.31 | 34.25 | 0.00 | 26 | 4351.79 |
| p(year+age)Ψ(Wind anom+age) | 4405.62 | 35.56 | 0.00 | 27 | 4351.06 |
| p(year+age)Ψ(Wind anom*age) | 4407.64 | 37.58 | 0.00 | 28 | 4351.03 |
| p(year+age)Ψ(year) | 4506.53 | 136.47 | 0.00 | 43 | 4419.11 |
| p(year+age) Ψ(.)) | 4572.18 | 202.12 | 0.00 | 25 | 4521.70 |

| **Institute** | **Acronym** | **Country** | **Model** |
| --- | --- | --- | --- |
| Centro Euro-Mediterraneo per i Cambiamenti Climatici | CMCC | Central Europe | CMCC-CM |
| Centre National de Recherches Meteorologiques | CNRM-CERFACS | Central Europe | CNRM-CM5 |
| Commonwealth Scientific and Industrial Research Organisation | CSIRO-BOM | Australia | Access 1.0 |
| Institute for Numerical Mathematics | INM | Russia | INM-CM4 |
| Max Planck Institute | MPI-M | Germany | MPI-ESM-MR |
| National Aeronautics and Space Administration | NASA-GISS | U.S.A. | GISS-E2 |
| National Center for Atmospheric Research | NCAR | U.S.A. | CCSM4 |
| Norwegian Climate Center | NCC | Norway | NOR-ESM-1 |

Table S3. List of institutes that developed CMIP5 General Circulation Models used in this paper.
